# Magnetic resonance imaging findings in bipartite medial cuneiform – a potential pitfall in diagnosis of midfoot injuries: a case series

**DOI:** 10.1186/1752-1947-2-272

**Published:** 2008-08-13

**Authors:** Ilan Elias, Sachin Dheer, Adam C Zoga, Steven M Raikin, William B Morrison

**Affiliations:** 1 Department of Orthopaedic Surgery, Rothman Institute, Thomas Jefferson University Hospital, 925 Chestnut Street, Philadelphia, PA, 19107, USA; 2 Department of Radiology, Division of Musculoskeletal Imaging, Thomas Jefferson University Hospital, 132 S. 10th St. Suite 1079a, Philadelphia, PA, USA

## Abstract

**Introduction:**

The bipartite medial cuneiform is an uncommon developmental osseous variant in the midfoot. To our knowledge, Magnetic Resonance Imaging (MRI) characteristics of a non-symptomatic bipartite medial cuneiform have not been described in the orthopaedic literature. It is important for orthopaedic foot and ankle surgeons, musculoskeletal radiologists, and for podiatrists to identify this osseous variant as it may be mistakenly diagnosed as a fracture or not recognized as a source of non-traumatic or traumatic foot pain, which may sometimes even require surgical treatment.

**Case presentations:**

In this report, we describe the characteristics of three cases of bipartite medial cuneiform on Magnetic Resonance Imaging and contrast its appearance to that of a medial cuneiform fracture.

**Conclusion:**

A bipartite medial cuneiform is a rare developmental anomaly of the midfoot and may be the source of midfoot pain. Knowledge about its characteristic appearance on magnetic resonance imaging is important because it is a potential pitfall in diagnosis of midfoot injuries.

## Introduction

**Figure 1:**
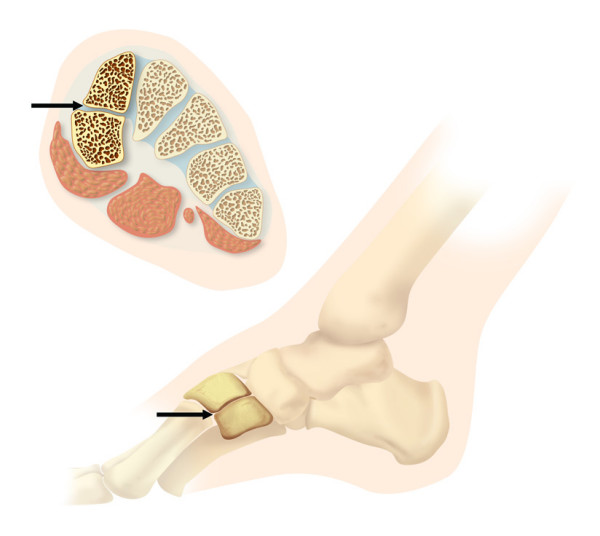
Drawing shows an axial and sagittal configuration of the bipartite medial cuneiform. Typically, there is a horizontal joint space between both cuneiform counterparts seen in the axial and sagittal (arrows) sections.

Originally described in an anthropologic population study in 1942, bipartite medial cuneiforms are an uncommon tarsal developmental variant (Figure 1) at the Lisfranc joint line occurring in approximately 0.3% of individuals [1]. There has been no description of MRI features of bipartite medial cuneiforms in the orthopaedic surgery literature. Nevertheless, identifying a bipartite medial cuneiform and differentiating it from a fracture is important. Orthopaedic foot and ankle surgeons, musculoskeletal radiologists and podiatrists should be aware of this osseous variation as it may be mistakenly diagnosed as a fracture and recognize that a bipartite medial cuneiform may be a cause of a non-traumatic or traumatic midfoot pain that may sometimes even require surgical treatment [2,3]. In this report, we describe the characteristics of three cases of bipartite medial cuneiform on Magnetic Resonance Imaging (MRI) and contrast its appearance to that of a medial cuneiform fracture.

## Case presentations

### Case 1: Bipartite medial cuneiform

**Figure 2:**
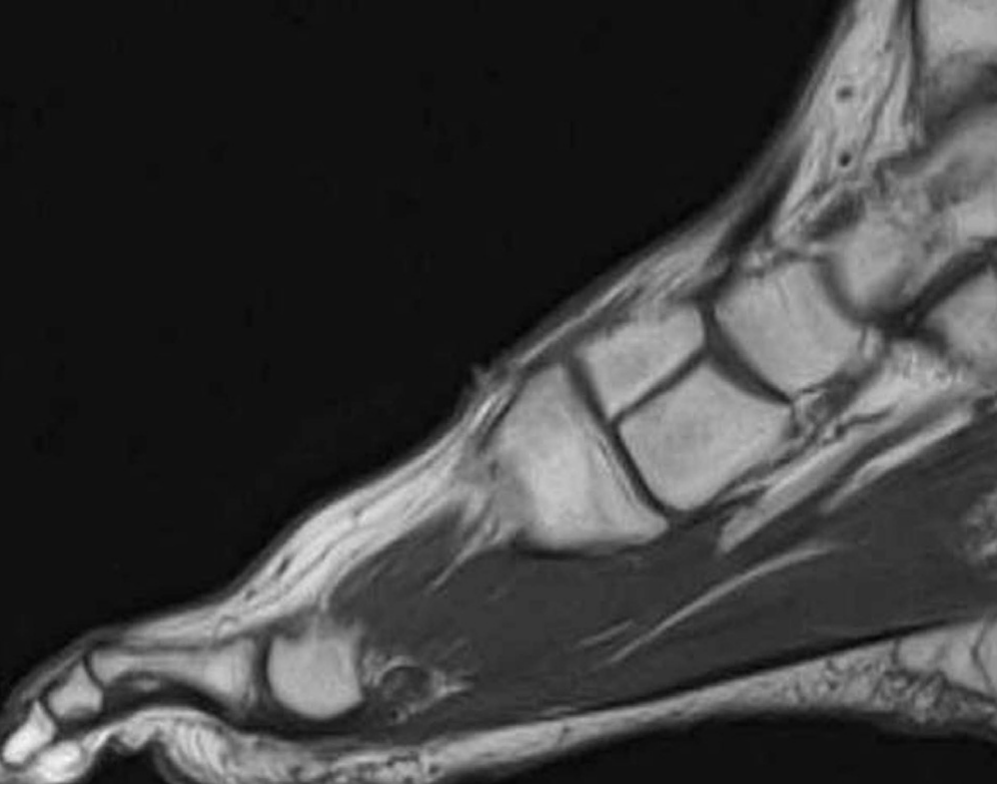
Sagittal T1 weighted spin echo MR image (TR/TE = 500/12 ms) demonstrates a typical bipartite medial cuneiform noted in case #1.

A 59-year-old male with chronic lateral ankle pain was referred for an MRI of the ankle by his podiatrist. The MRI demonstrated a split type tear of the peroneus brevis tendon and a plantar calcaneal heel spur. Incidental note was made of a bipartite medial cuneiform (Figure 2). The patient had no symptoms in the region of the medial midfoot. He was prescribed partial weight bearing, bracing and physical therapy, with partial relief and had no imaging follow-up.

### Case 2: Bipartite medial cuneiform

**Figure 3:**
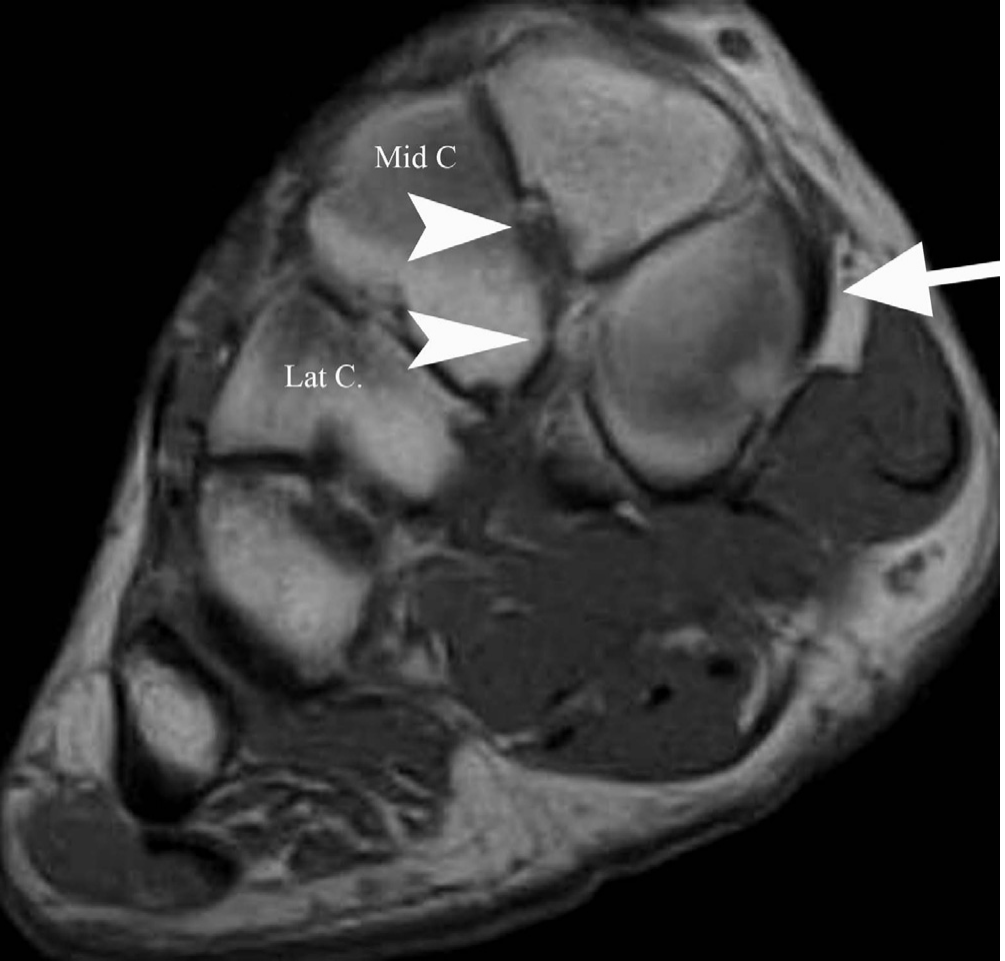
**Short axis (coronal) proton density spin echo MR image**. (TR/TE = 1800/12 ms) at the level of the midfoot shows a smooth, horizontally oriented, well corticated cleavage of the medial cuneiforms noted in case #2. The posterior tibial tendon inserts on the medial aspect of the plantar segment (arrow) and the dorsal and plantar bundles of the Lisfranc ligament each insert on the respective portions of the medial cuneiform (both arrowheads). Mid C = middle cuneiform; Lat C = lateral cuneiform.

A 34-year-old male long-distance runner with lateral metatarsal pain was referred for an MRI of the foot by his orthopaedic surgeon. The MRI demonstrated bone marrow edema within the fourth metatarsal shaft and an incomplete fracture of the proximal fourth metatarsal shaft. A bipartite medial cuneiform was incidentally noted (Figure 3). No symptoms were present in the region of the midfoot. The patient has been prescribed non-weight bearing treatment, without MR imaging follow-up.

### Case 3: Fractured medial cuneiform

**Figure 4:**
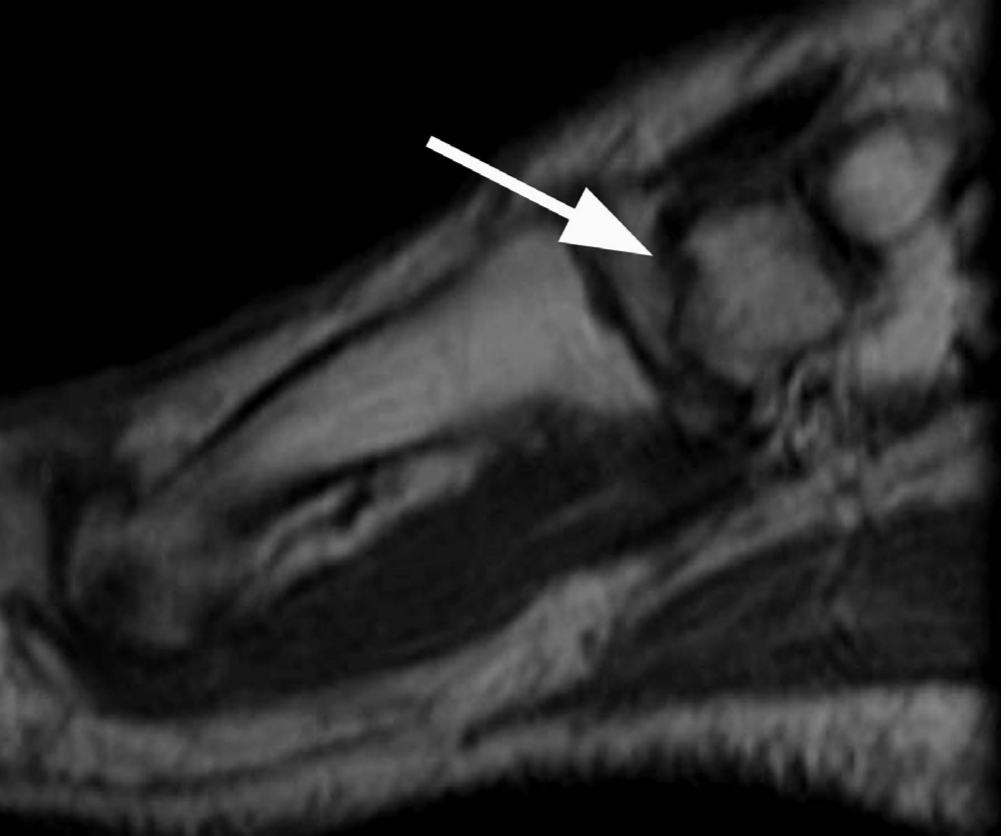
Sagittal T1 spin echo MR image (TR/TE = 700/9 ms) of case #3 demonstrates an oblique, hypointense fracture line (arrow) through the medial cuneiform, without significant displacement.

A 44-year-old male with a history of multiple sclerosis presented to his orthopedist with left midfoot pain following a motorcycle accident. Radiographs performed at this time were interpreted as a minimally displaced medial cuneiform fracture and therapy with a short leg, non-weight bearing cast for 6 weeks was suggested. The patient continued to bear weight and experience pain, then sought a second opinion, at which time an MRI examination of the ankle was performed (Figure 4). Following the MRI, the patient was advised to stop weight bearing, which resulted in a resolution of his symptoms. No further MR imaging follow-up was obtained in this case.

### Case 4: Bipartite medial cuneiform developing arthritis

**Figure 5:**
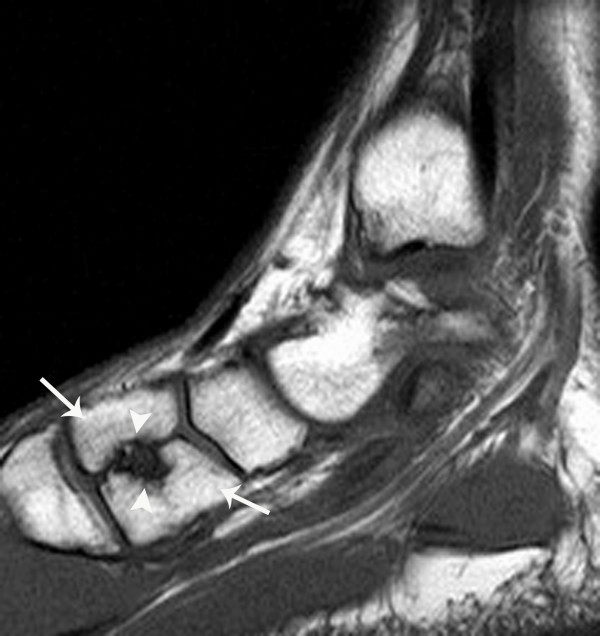
Sagittal T1 weighted spin echo (TR/TE = 700/9 ms) MR image of a partially bipartite medial cuneiform with early osteoarthritis of case #4. The cuneiform clearly includes two morphologic fragments (arrows) and there are erosive changes (arrowheads) about the cleavage plane suggesting partial fusion and some instability or motion between fragments.

**Figure 6:**
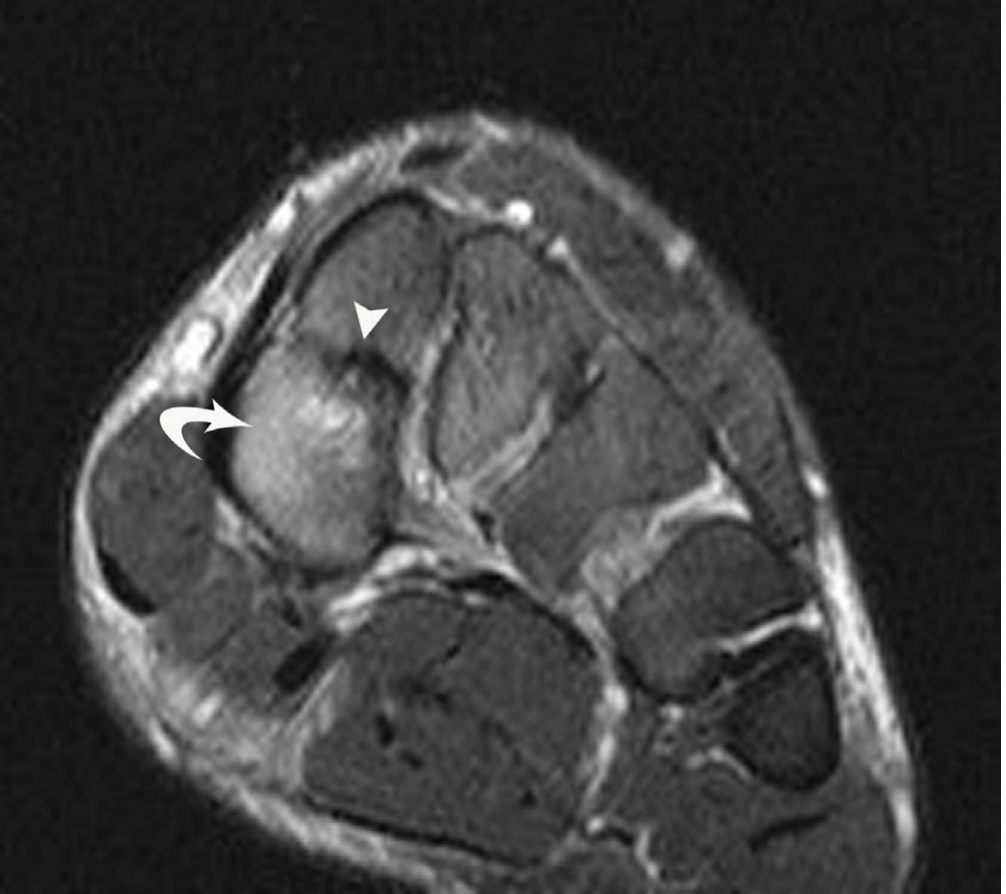
**Coronal T2 weighted fast spin echo fat suppressed image**. (TR/TE = 3000/46 ms) of case #4 shows a bipartite medial cuneiform, likely with a fibrous coalition of the fragments and osseous erosion or cystic change (arrowheads) as well as reactive bone marrow edema (curved arrow).

A 36-year-old male presented with worsened chronic medial foot pain after a supination injury. Because of concern for an occult fracture, an MRI examination dedicated to the ankle and midfoot was performed. A bipartite medial cuneiform was incidentally noted with a fibrous coalition of the fragments and early subchondral cystic change spanning the segmentation suggesting abnormal motion and developed degenerative arthritis (Figures 5 and 6).

## Discussion

The bipartite medial cuneiform was originally described by Barlow in 1942 [1]. Subsequent cadaveric study demonstrated that the incidence of this variant is between 0.3% and 2.4% [4]. In cases of medial cuneiform bipartition, the cuneiform bone is divided horizontally by a synchondrosis, and the plantar segment is larger. Portions of the posterior tibial and peroneus longus tendons attach to the proximal inferomedial and distal inferolateral portions of the plantar segment. The anterior tibial tendon inserts on the proximal superomedial dorsal segment and the dorsal and plantar bundles of the Lisfranc ligament attach to the respective portions of the medial cuneiform [5].

It is believed that the normal medial cuneiform develops from one primary ossification center. In the setting of two primary ossification centers, these may fail to fuse, resulting in bipartition. Ossification of the lateral cuneiform begins in the first year of life, followed by the medial and middle cuneiforms in the second and third years, respectively [5]. Most cases of a bipartite medial cuneiform have been reported incidentally; however, some authors have identified and successfully treated chronic foot pain believed to be associated with a bipartite cuneiform. In two patients, one of whom was a marathon runner and the other a military recruit, excision of the dorsal segment and steroid injection into the joint have been reported as successful treatments [2,6]. A third patient with chronic foot pain after remote trauma has reportedly been successfully treated with fixation using a trans-cortical screw [3].

A fracture of the synchondrosis between the two segments of a bipartite medial cuneiform has also been reported in a pediatric patient [7]. From an imaging standpoint, it is important to identify a bipartite medial cuneiform and differentiate it from a fracture. A bipartite medial cuneiform should demonstrate smooth, well corticated margins. The two portions of the bipartite cuneiforms together are usually larger than the expected normal, or fractured medial cuneiform as for example seen in case #3. Also, we found in all cases of a bipartite medial cuneiform that the proximal articular surface of the first metatarsal bone was larger than usual.

**Figure 7:**
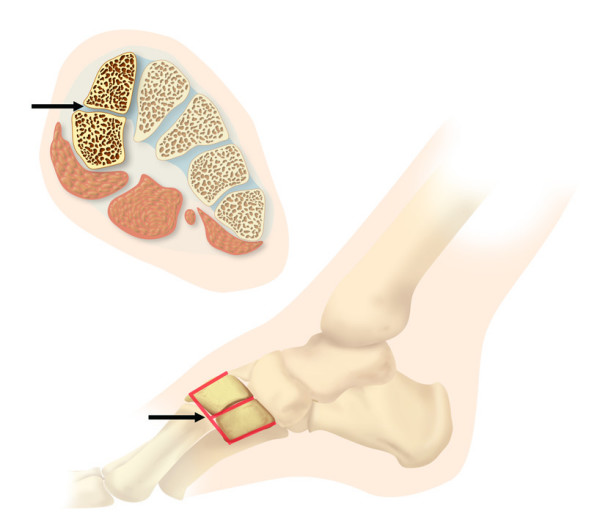
Drawing shows an axial and sagittal configuration of the bipartite medial cuneiform. On sagittal images, there is a typical '**E**'-sign (arrow) within the two bipartite medial cuneiform bones.

An asymptomatic bipartite medial cuneiform should not have associated bone marrow edema, as a fracture might. Additionally, the cleavage plane between the two fractured portions of the cuneiform would typically be irregular, not smooth, as in the case of a bipartition. All cases of bipartition indicate that the cleavage plane between the two portions of the bone is horizontally oriented (along the long axis of the foot), which would be atypical for a fracture. In fact, in all three bipartite cases in our series, we found well-defined joint spaces between the head of the first metatarsal and the distal aspect of the two medial bipartite cuneiform bones as well as a well-defined horizontal joint space between the two bipartites. These joint spaces between the three bones demonstrate a unique 'E' joint space configuration on sagittal MR images, what we define as E-sign (Fig. 7). The E-sign is useful to identify a bipartite medial cuneiform on MRI. The MRI findings of a symptomatic bipartite medial cuneiform have been recently reported [8]. Two of our cases #1 and #2 did not demonstrate any subchondral bone marrow edema, suggesting the absence of altered biomechanics [9] or increased stress across the joint. As such, the authors believe that they were true incidental findings. In cases of symptomatic bipartition, evidence of symptoms may be found with the presence of bone marrow edema or productive osseous change centered around the joint as we have found in our third bipartite case #4.

## Conclusion

A bipartite medial cuneiform is a rare developmental anomaly of the midfoot and has a characteristic appearance on MRI (E-Sign). Knowledge of the presence and appearance of this osseous variant is important in being able to identify this entity and to differentiate it from a fracture because this may potentially be a pitfall in diagnosis of midfoot injuries. Even in the absence of a fracture, a bipartite medial cuneiform may be the source of midfoot pain, which can be treated with various techniques, including surgery [3].

## Competing interests

The authors declare that they have no competing interests.

## Authors' contributions

IE and SD prepared the manuscript; ACZ and WBM reviewed the manuscript; all authors (IE, SD, AZ, SMR, and WBM) reviewed the patients' data and MRIs.

## Consent

This study was approved by the Institutional Review Board (IRB) of the Thomas Jefferson University Hospital. Written informed consent could not be obtained in these cases since the patients are untraceable. We believe this case series contains a worthwhile clinical lesson, which could not be as effectively made in any other way. We expect the patients not to object to the publication since every effort has been made so that they remain anonymous.
